# Impact of cardiac rehabilitation programs on left ventricular remodeling after acute myocardial infarction: Study Protocol Clinical Trial (SPIRIT Compliant)

**DOI:** 10.1097/MD.0000000000019759

**Published:** 2020-04-17

**Authors:** Mihaela Ghircau Susca, Roxana Hodas, Theodora Benedek, Imre Benedek, Monica Chitu, Diana Opincariu, Andreea Chiotoroiu, Ciprian Rezus

**Affiliations:** a University of Medicine, Pharmacy, Sciences and Technology of Targu Mures; b Clinic of Cardiology, University of Medicine, Pharmacy, Sciences and Technology of Targu Mures; c Department of Advanced Research in Multimodality Cardiovascular Imaging, Cardio Med Medical Center, Targu Mures; d University of Medicine and Pharmacy ‘Gr.T.Popa’, Iasi, Romania.

**Keywords:** acute myocardial infarction, cardiac rehabilitation, left ventricular remodeling, systemic inflammation

## Abstract

**Introduction:**

While the role of early mobilization in the immediate postinfarction period has been well demonstrated, little is known in present about the link between early mobilization and reduction of systemic inflammation. At the same time, the impact of early mobilization on regression of left ventricular remodeling has not been elucidated so far.

**Material and methods:**

Here we present the study protocol of the REHAB trial, a clinical descriptive, prospective study, conducted in a single-center, with the purpose to analyze the impact of early mobilization in reducing left ventricular remodeling, the complication rates and mortality in patients who had suffered a recent acute myocardial infarction (AMI). At the same time, the study aims to demonstrate the contribution of early mobilization to reduction of systemic inflammation, thus reducing the inflammation-mediated ventricular remodeling. 100 patients with AMI in the last 12 hours, and successful revascularization of the culprit artery within the first 12 hours after the onset of symptoms in ST-segment elevation acute myocardial infarction or within first 48 hours in non ST-segment elevation AMI will be enrolled in the study. Based on the moment of mobilization after AMI patients will be distributed in 2 groups: group 1 – patients with early mobilization (<2 days after the onset of symptoms) and; group 2 – subjects with delayed mobilization after AMI (>2 days after the onset of symptoms). Study outcomes will consist in the impact of early mobilization after AMI on the ventricular remodeling in the post-infarction period, as assessed by cardiac magnetic resonance imaging, the rate of in-hospital mortality, the rate of repeated revascularization or MACE and the effect of early mobilization on systemic inflammation in the immediate postinfarction phase.

**Conclusion:**

In conclusion, REHAB will be the first trial that will elucidate the impact of early mobilization in the first period after AMI, as a first step of a complex cardiac rehabilitation program, to reduce systemic inflammation and prevent deleterious ventricular remodeling in patients who suffered a recent AMI.

## 1 Introduction

### 1.1 Background and rationale

As a severe form of coronary heart disease, acute myocardial infarction (AMI) involves a significant burden in terms of disability, especially in the increasing population of elderly patients.^[1,2]^ Impressive progress of diagnostic and therapeutic procedures in the last years significantly reduced the AMI-related mortality. As a consequence, rehabilitation of AMI survivors has become a real public health issue and a major challenge to be solved.^[3,4]^ In this context, both health care systems and general population have started to become aware of the fact that the current approach, mainly involving interventional and pharmacological treatments, is neither effective nor sufficient.^[5,6]^ As patients with recent AMI deserve special attention, structured multifaced and multidisciplinary interventions for assessment and management of cardiovascular (CV) risk factors, guidance on physical activity and psychosocial sustenance^[7,8]^ were developed and implemented as case-management models of cardiac rehabilitation (CR) programs.^[9–11]^

Divided in 3 phases: inpatient, outpatient, and long-term interventions, CR aims to improve functional capacity and recovery, prevent disability and restore the quality of life.^[12]^ Along with patient physical reintegration, CR programs involve preventive strategies for subsequent CV events, death or hospitalization from cardiac causes.^[13]^ While the objectives are identical, settings differ according to local or national protocols and experiences, including residential, ambulatory community, or home-based strategies.^[14]^

One of the most important predictors of long-term evolution after an AMI is represented by the left ventricular (LV) remodeling. Postinfarction LV represents a maladaptive and dynamic process resulting from the complex interaction between the size of the infarcted area, genetic predisposition and inflammation, in which systemic inflammation plays a crucial role. This phenomenon is associated with mechanical and biochemical changes, leading to arrhythmia, ventricular dysfunction and heart failure on the long term.^[15–18]^ A large variety of biomarkers have proven their prognostic role in LV remodeling, being well associated with inflammation and microcirculatory dysfunction (NT-proB-type natriuretic peptide, high-sensitive cardiac troponin T, aspartate aminotransferase, alanine aminotransferase, high sensitive C-reactive protein [hs-CRP], and lactate dehydrogenase).^[19]^

Among inflammatory biomarkers whose maintenance at a high serum level has been proved to be prognostic for ventricular remodeling, interleukin-6 (IL-6), tumor necrosis factor-α, and interleukin-1β are the most widely studied, and may serve as prognostic markers for the development of postinfarction heart failure. At the same time, it has been hypothesized that specific CD11/CD18 integrin receptors, complement cascade activators, complement component 1, complement component 5 or P-selectin can be used to form antibodies that would block the inflammation, however the results of such studies are still un-conclusive.^[20–23]^

In a series of non-pharmacological therapeutic strategies concerning the inflammatory component of atherosclerotic disease, exercise training proved to be the most outstanding.^[24]^ The success of regular exercise in reducing the risk of coronary disease development and complications was strongly associated with the reduction of C-reactive protein (CRP) and fibrinogen levels.^[25]^ Moreover, Church et al found an inverse correlation between cardiorespiratory exercise and systemic level of CRP, an inflammation-related biomarker validated as a significant predictor for the development of an AMI.^[25,26]^

According to current ESC and ACC/AHA guidelines for the management of patients with ST-segment elevation acute myocardial infarction (STEMI), mobilization should be performed in the first 12 and 24 hours following an uncomplicated acute AMI (level of evidence C).^[26,27]^ While the role of early mobilization in the immediate postinfarction period has been well demonstrated, little is known in present about the link between early mobilization and reduction of systemic inflammation. At the same time, the impact of early mobilization on regression of LV remodeling has not been elucidated so far.

### 1.2 Study objectives

The *primary objective* of the study is to evaluate the impact of early mobilization after AMI on the ventricular remodeling in the post-infarction period, as assessed by cardiac magnetic resonance (CMR) imaging.

The *secondary objectives* include

(1) to investigate the effect of early mobilization on systemic inflammation in the immediate postinfarction phase
(2) to investigate the impact of early mobilization on in-hospital mortality and the role of early mobilization in reducing in-hospital complications in patients suffering an AMI.

## 2 Methods/design

### 2.1 Study design

This is a prospective, non-randomized, cohort study, carried out in a single-center, which aims to assess the link between early mobilization after AMI, systemic inflammation, and LV remodeling in patients with recent STEMI.

### 2.2 Ethics approval

The clinical study has the approval of the local Ethics Committee for Scientific Research of the University of Medicine and Pharmacy of Tirgu-Mures (certificate of approval: 348/13.12.2017) and the Ethics Committee for Scientific Research of the Cardio Med Medical Center (certificate of approval: 30/28.12.2017). All study procedures will be conducted according to the Declaration of Helsinki and each subject will provide signed written informed consent before randomization process.

### 2.3 Study population

The study will be a single-center, observational, non-randomized study including 100 patients with AMI, presenting with either STEMI or non ST-segment elevation AMI (NSTEMI).

Inclusion criteria:

- Patients with AMI in the last 12 hours.
- Successful revascularization of the culprit artery within the first 12 hours after the onset of symptoms in STEMI or within first 48 hours in NSTEMI (according to the risk class).
- Signed written informed consent.

Exclusion criteria:

- Patient refusal;
- Any condition that would contraindicate CMR examination;
- Women during pregnancy or lactation period;
- Women able to procreate without any contraceptive usage;
- Chronic kidney disease (glomerular filtration rate <60 ml/min/1.73m^2^) or acute renal injury that requires hemodialysis;
- Any type of neoplasia documented in the last 3 years before randomization;
- Expectation of life <1 year.

### 2.4 Study settings

The study will be carried out in the Center of Advanced Research in Multimodality Cardiac Imaging of the Cardio Med Medical Center, in Targu Mures, Romania, and funding will be provided by the European Union and the Government of Romania through the Ministry of European Funds, accessed via research grant number 103545/2016 - “High performance multimodal MRI/CT imaging platform, for applications in computational medicine, nanoparticles and hybrid imaging for the research of atherothrombotic disorders - CARDIO IMAGE” - (contract number 43/05.09.2016).

### 2.5 Study groups

This study will enroll 100 patients with AMI meeting the inclusion criteria, who will be distributed in two groups: group 1 – patients with early mobilization (<2 days after the onset of symptoms) and; group 2 – subjects with delayed mobilization after AMI (>2 days after the onset of symptoms).

### 2.6 Study procedures and outcome assessment

- Medical records, physical exam, laboratory analysis (complete blood count [CBC], biochemistry, serum levels of hs-CRP, matrix metalloproteinase, IL-6, NT-pro-BNP);
- Electrocardiography
- Transthoracic echocardiography for assessment of LV systolic and diastolic performance, speckle tracking echocardiography, Dobutamine viability test
- Late gadolinium enhancement CMR for evaluation of ventricular function and remodeling, extent of myocardial scar and transmurality index.

#### 2.6.1 Biomarkers assays

Routine biochemistry and CBC analysis will be performed at the main laboratory of the Emergency Clinical County Hospital of Tirgu-Mures, immediately after randomization, during the acute phase of infarction. Inflammatory status (hs-CRP levels) will be evaluated at 7 days after the acute cardiac event via immunoturbidimetric assay. Serum levels of matrix metalloproteinase 9 and IL-6 will be evaluated with enzyme linked immunosorbent assay. Serum inflammatory biomarkers will be analyzed in Advanced Medical and Pharmaceutical Research Center of the University of Medicine and Pharmacy Tirgu-Mures. Serum levels of NT-proBNP will be determined at randomization, at the main laboratory of the Emergency Clinical County Hospital of Tirgu-Mures by electrogenerated chemiluminescence.

#### 2.6.2 Transthoracic echocardiography

Transthoracic echocardiographic assessment will be performed at first visit in all patients, in the first days during the hospitalization for the acute coronary event. Speckle tracking technology will be used for evaluation of ventricular volumes and wall motion analysis, using a Vivid E9 ultrasound system (General Electric Vingmed Ultrasound, Horten, Norway).

#### 2.6.3 Late gadolinium-enhancement cardiac magnetic resonance (LGE-CMR)

All CMR images will be acquired with the use of a 1.5T Siemens Magnetom Aera equipment. The LGE-CMR will be used to quantify the infarct size, the extent of myocardial necrosis and the transmurality index, 10 minutes after gadolinium intravenous injection. Imaging data post-processing will be conducted with the use of Medis Q Mass 8.1 software (Medis, Leiden, the Netherlands), which involves manually tracing of the epicardial and endocardial borders, while setting the threshold for hyper-enhancement in the acquired image sequence.

### 2.7 Study timeline

REHAB study will be conducted from October 2019 to October 2020.

### 2.8 Outcomes

The *primary outcome* of the study will consist in the impact of early mobilization after AMI on the ventricular remodeling in the post-infarction period, as assessed by CMR imaging.

*Secondary outcomes* of the study will be represented by rate of in-hospital mortality and the rate of repeated revascularization or MACE (including CV death or stroke) in patients with early mobilization as compared to those with delayed mobilization, and the effect of early mobilization on systemic inflammation in the immediate postinfarction phase.

### 2.9 Participation timeline

Baseline (day 0):

- Achieve written informed consent form all patients.
- Check all inclusion/exclusion criteria.
- Record demographic information, medical records, CV risk factors
- Perform and record physical examination and 12-lead electrocardiography (ECG).
- Laboratory analysis (CBC, routine biochemistry, inflammatory biomarkers, acute adhesion molecules).
- Transthoracic echocardiography / speckle tracking.

Visit 1 (day 7 / discharge from the hospital):

- hs-CRP assessment.

Visit 2 (month 1):

- LGE-CMR (myocardial fibrosis/scar, infarct size, transmurality, remodeling).

Visit 3,4,5 (month 3,6,9)

- Record results of physical exam, medical records, ECG.
- Transthoracic echocardiography / speckle tracking.

Final study visit (month 12)

- Record results of physical exam, medical records, ECG.
- Transthoracic echocardiography / speckle tracking.
- End-point assessment.

### 2.10 Sample size

The study will enroll 100 patients with AMI who presented within the first 12 hours after the onset of symptoms and underwent successful revascularization of the culprit artery within the recommended timeframe, with TIMI 3 flow. Patients will be divided into 2 groups on the basis of the time interval from admission to mobilization: early (<2 days) mobilization group, versus delayed (>2 days) mobilization group. The sample size was calculated using Stat Mate 2.0 software, which indicated that a sample size of 50 subjects per group has a 90% power to detect an increase in major adverse CV events rates proportion of 3.28, with a significance level (alpha) of 0.05 (2-tailed).

## 3 Discussions

This manuscript presents the protocol of a clinical descriptive, study, conducted in a single-center, with the purpose to analyze the impact of early mobilization in reducing LV remodeling, the complication rates and mortality in patients who had suffered a recent AMI. At the same time, the study aims to demonstrate the contribution of early mobilization to reduction of systemic inflammation, thus reducing the inflammation-mediated ventricular remodeling.

### 3.1 Cardiac rehabilitation after AMI

After 50 years from the first implementation of a post AMI CR program, the clinical benefits and cost-effectiveness of CR are now unequivocally demonstrated by clinical evidence, CR becoming an indispensable component of patient-oriented care.^[28,29]^ Published results have documented that CR improves patient outcomes, on a magnitude similar to the reduction in mortality and morbidity rates obtained by aspirin therapy or percutaneous coronary intervention implementation, and probably with similar cost-to-benefit ratios.^[30]^

Supported by systematic reviews and meta-analyses documenting a reduced mortality,^[31]^ at this moment CR after AMI is a Class I recommendation from both European and American guidelines.^[28]^ Unfortunately, despite the body of professional recommendations indicating CR as the most important evidence-based intervention for secondary prevention after AMI, CR programs integration into daily practice proves to be a challenge. In despite of the availability of suitable CR programs, many studies demonstrate currently that only 25% of suitable patients are referred^[32]^ or finally take up any form of CR,^[33]^ with <10% participation in elderly subjects. Moreover, even when implemented, most of CR programs rely only on short-term strategies, with 30% to 40% of patients discontinued CR after first 6 months, and up to 50% dropping out after 1 year.^[34]^ A series of research indicates the barriers that hold responsibility for the underuse of CR are generally centered around patient, healthcare provider, health system and community levels.^[35]^ Patient involvement seems to be particularly challenging, therefore implementation of novel strategies is urgently needed to address this problem, as this approach can minimize the probability of subsequent coronary events while maximizing functional capacity.^[36]^ Home-based programs, community-based group strategies or internet-based programs may represent alternative approaches to structured supervised CR providing risk modification, education and guidance.^[37–40]^

### 3.2 Ventricular remodeling after acute myocardial infarction

Progression of the LV remodeling leads to alterations in cardiac geometry, function and structure of the myocardium. This affects the ventricular contraction that becomes asymmetric. Therefore, LV dilates, the cardiac volumes increases and the stroke volume decreases.^[15,18,41,42]^

One of the most important determinants of the LV remodeling is the infarct size. However, in some situations, although the infarcted area has been enlarged, myocardial reparative response can prevent the development of LV remodeling.^[15,42,43]^ Studies proved that initiation of the LV remodeling process does not correlate only with the infarcted size. LV remodeling depends also on inflammatory response that involves local chemokine production, necrotic tissues removal and extracellular matrix formation.^[15,18,41–44]^ Several recent studies have proved that miRNAs play also an important role in LV remodeling. The reduced expression of miRNA after AMI correlates with a proliferation of cytokines to the fibroblast line, thus with cardiac fibrosis and consequently the reduction of myocardial vascularization favoring LV remodeling.^[45]^

### 3.3 Impact of early mobilization on systemic inflammatory response after AMI

The inflammatory response after AMI plays an important role in cardiac repair especially in preventing the occurrence of adverse LV remodeling and the onset of heart failure.^[45–46]^ After AMI, the sudden death of cardiomyocytes occurs, leading to the emergence of a pro-inflammatory response necessary for the necrotic cell clearance cells from the infarcted area. Paradoxically, myocardial reperfusion therapy leads to the aggravation of this inflammatory process. Thus, overexpression of proteases – matrix metalloprotease MMP2 and MMP9 and an impairment of autophagosome clearance occurs, leading to activation of the RISK pathways. The most frequent outcomes are associated with cardiac dilatation, infarct area expansion and cardiac fibrosis.^[46–50]^

A large multiple cytoplasmic complex is formed that mediates activation of pro-inflammatory cells (IL-1, IL-6, IL-18, tumor necrosis factor) with critical role in amplifying the pro-inflammatory response. Thus, the activation of natural killer cells and inflammasomes represents the second stage of the cytokine response to AMI. Inflammasomes are cytoplasmatic complexes that typically contain one of the nod-like receptor family proteins capable to recognize and induce the inflammatory response after AMI.^[15,46,51]^ Endothelial cells are one of the major sources of pro-inflammatory chemokines responsible for molecular adhesion to the endothelial surface. With an important role in the recruitment of neutrophils, monocytes and lymphocytes in the infarcted area, endothelial cells increase reactive oxygen species production by activating various signaling pathways, which involve additional secretion of chemokines and cytokines.^[15,46,47]^

In the first minutes after AMI, blood cytokines invade the infarcted area through the CCL2 / C-C chemokine receptor type 2 signal pathway. Early reperfusion of the myocardial tissue reduces the number of cytokines accumulated in the infarcted area by stimulating the immune reaction and thus is necessary for a favorable cardiac response after AMI.^[52,53]^ On the other hand, an insufficient repair response may be responsible for cardiac rupture or LV aneurysm. In conclusion, mechanical properties of scar tissue are the determinants of AMI-related outcomes.^[54]^

### 3.4 Inflammatory biomarkers and remodeling after AMI

In the acute phase of AMI, maintenance of the pro-inflammatory - anti-inflammatory balance is dependent on the multifactorial interaction between cellular elements (myocytes, fibroblasts, interstitial) and immunological mechanisms mediated by lymphocytes, fibroblasts, dendritic cells.^[47,55,56]^

The cytokines released by the inflammatory cells have various actions on the myocardium as follows: activation of proteinases, acceleration of myocyte apoptosis with reduced contractility, increase of intercellular matrix, activation of fibroblasts. This effects lead to hypertrophy of non-infarcted myocardial segments, thinning of the necrosis area, myocardium pathological remodeling profile, which is aggravated by the persistence of the proinflammatory status.^[57–59]^

The complexity of the chronic inflammatory phenomenon after AMI requires a multifactorial approach against both cell dynamics, humoral mechanisms and chemical cellular reactions of mitochondrial destructive type, oxidative stress and calcium loading, which is why the therapeutic approach must be anti-inflammatory, mitochondrial and endothelial protective.^[60–62]^

### 3.5 The effect of cardiac rehabilitation programs on inflammatory response and ventricular remodeling after AMI

The severe tissue damage caused by AMI involves a generalized inflammatory reaction in the early period, orchestrating healing and recovery of the heart.^[63,64]^ However, in certain circumstances excessive inflammatory response may cause further myocardial impairment and excessive fibrosis, leading to heart failure.^[65,66]^ A three-month program of CR reported a favorable effect of early CR on serum levels of hsCRP, independent of statin therapy and weight reduction. In this study CR produced similar reduction in hsCRP levels as therapy with statins, a 50% reduction of hsCRP levels being obtained in patients treated with statins coupled with CR and exercise training. Moreover, similar reduction in hsCRP levels were observed regardless of variation in weight, suggesting that exercise training play a prominent role in inflammation reduction.^[25]^ The positive impact of exercise training on the reduction of IL-6 and tumor necrosis factor α soluble receptor 1 levels was demonstrated in a group of patients with moderately severe and severe chronic heart failure and known ischemic heart disease.^[67]^ The anti-inflammatory cytokine IL-10 primarily inhibits the release of tumor necrosis factor-α. Exercise induces a cascade of cytokine inhibitors, but also the protective role of IL-10.^[68]^ However, the impact of early rehabilitation programs after AMI is not elucidated yet, while most of the studies concern the effects of physical rehabilitation on inflammatory status in chronic stable disease. In a three-week CR study conducted in early post-AMI period, while passive recovery with optimal medical treatment led to a slow inflammatory regression, physical training of moderate intensity proved an additional anti-inflammatory effect. Beside the impact on inflammatory markers, CR involved a significant improvement in terms of metabolic risk profile.^[25]^ Changes in hsCRP levels were correlated with exercise training and the degree of BMI. Since adipose tissue consist the source of IL-6, a precursor of CRP, it could be presumed that CR could directly influence the fatty cell metabolism, causing suppression of the inflammatory pathway. These results suggest that overweight and obese patients, presenting a high-risk group in secondary CV prevention due to their inclination towards the metabolic syndrome, could particularly benefit from early CR after AMI.^[25]^ Even if effects of CR on IL-10 levels are poorly investigated, Smith et al reported a 36% increase of Il-10 levels in patients performing a 6-months CR.^[69]^ IL-8 is considered one of the leading promoters of atherosclerosis in diabetic and obese subjects^[70,71]^ and presents significantly decreased plasma concentrations in patients who followed a CR program as compared with a control group.^[72]^ Beside its benefits in terms of inflammatory status, at this moment growing clinical consensus indicate CR beneficial impact on course of post-AMI cardiac remodeling process.^[73,74]^ In AMI survivors presenting left ventricle (LV) systolic disfunction CR has been recommended to the medical therapy in order to prevent the progression of LV systolic impairment,^[75,76]^ as physical exercise increase myocardial perfusion independent of coronary lesions,^[77]^ a fact that may induce a consistent recovery of regional and global LV contractility.^[78]^ Moreover, compelling evidence showed that CR implementation in post-AMI patients induce a favorable course of LV remodeling process,^[79]^ presenting pro-angiogenic effects, as inadequate angiogenesis represents a critical cornerstone process in the development of maladaptive remodeling, promoting the transition from cardiac hypertrophy to dilatation and dysfunction.^[80–83]^ Although majority of research results proved a positive influence of CR in post-AMI patients, improving adverse remodeling process and cardiac function, the proper CR intensity, duration and time to start are yet to be optimized.^[78]^

*In conclusion*, the primary contribution of the REHAB trial will be to elucidate the impact of early mobilization in the first period after AMI, as a first step of a complex CR program, to reduce systemic inflammation and prevent deleterious ventricular remodeling in patients who suffered a recent AMI.

## Author contributions

All authors have been involved in all stages of the study design and have participated in writing the protocol. Submission to ethical committee was done by Ghircau Susca Mihaela.

Ghircau Susca Mihaela, Opincariu Diana, and Theodora Benedek will be involved in trial statistical analysis and interpretation. All the authors approved the final manuscript.
